# Engage for equity plus: Transforming academic health centers to sustain patient/community engaged research structures, policies, and practices

**DOI:** 10.1017/cts.2025.51

**Published:** 2025-03-19

**Authors:** Shannon Sanchez-Youngman, Belkis Jacquez, Prajakta Adsul, Elizabeth Dickson, Tabia Henry Akintobi, LaShawn Hoffman, Lisa G. Rosas, Starla Gay, Jason A. Mendoza, Diane Mapes, John Oetzel, Donald Nease, Nina Wallerstein

**Affiliations:** 1 University of New Mexico, College of Population Health, Albuquerque, NM, USA; 2 Department of Internal Medicine, School of Medicine, University of New Mexico Health Sciences Center, Albuquerque, NM, USA; 3 Cancer Control and Population Sciences Research Program, Comprehensive Cancer Center, University of New Mexico, Albuquerque, NM, USA; 4 Morehouse School of Medicine, Prevention Research Center, Atlanta, GA, USA; 5 Chair, Community Coalition Board, Prevention Research Center, Principal, Hoffman and Associates, Morehouse School of Medicine, Atlanta, GA, USA; 6 Department of Epidemiology and Population Health, Office of Community Engagement, Stanford University School of Medicine, Stanford, CA, USA; 7 Founder, Black Ladies Advocating for Cancer Care, Patient Advocate Member of Champion Team, Stanford University, Stanford, CA, USA; 8 University of Washington School of Medicine, Fred Hutchinson Cancer Center, Seattle, WA, USA; 9 Founding Advocate, Lobular Breast Cancer Alliance, Patient Advocate Member of Champion Team, Seattle, WA, USA; 10 The University of Waikato, New Zealand School of Management and Marketing, Hamilton, New Zealand; 11 University of Colorado, Family Medicine, Aurora, CO, USA

**Keywords:** Patient and community engaged research, community-based participatory research, institutional change, collective reflection, academic health center, structural barriers, champion teams

## Abstract

**Introduction:**

Community-based participatory research (CBPR) and patient/ community engaged research (P/CEnR) are shown to be effective approaches that improve health inequities, particularly among disadvantaged populations. While the science of CBPR demonstrates promising partnering practices that lead to effective interventions, there are institutional and structural barriers to creating and sustaining patient/community research within academic health centers (AHCs). As the field matures, there is a growing need to enhance patient/community leadership so that communities can set their own research agendas and priorities.

**Methods:**

Engage for Equity PLUS sought to address these challenges by implementing an engagement intervention aimed at transforming AHCs through supporting champion teams of academic, community, and patient partners to strengthen research infrastructures for P/CEnR. This paper uses a qualitative, case study analysis to describe how E2PLUS enabled champion teams at Stanford School of Medicine, Fred Hutchinson/University of Washington Cancer Consortium, and Morehouse School of Medicine to pursue institutional change strategies through coaching, workshops, contextual data analysis, and a community of practice.

**Results:**

This paper describes key themes of how E2Plus helped identify targets of change by a) using institutional data collection as core to generating critical consciousness of contextual conditions; b) implementing feasible E2PLUS strategies to leverage conditions for catalyzing a champion team for advocacy and achievable actions; c) identifying the critical role of patients/community members in stimulating change; and d) the role of continual collective reflection.

**Conclusion:**

We discuss the overall implications for E2 PLUS for other AHCs working toward sustainable community/patient engaged research policies and practices.

## Introduction

The health science field increasingly recognizes the value of community-based participatory research (CBPR) and patient/community engaged research (P/CEnR) in developing effective interventions that address root causes of disease [1–5]. While CBPR/CEnR encompasses a wide spectrum of projects, growing evidence shows these approaches share common values and practices that disrupt traditional knowledge production and catalyze academic and community partners to challenge structural and social conditions associated with health and racial inequities [6–14]. The acceptance of the field is also demonstrated by academic funding mechanisms that increasingly call for the inclusion of CBPR/CEnR approaches in health research.

This shift of CBPR into the mainstream has been driven, at least in part, by growing evidence which suggests that CBPR/CEnR relational and structural processes lead to effective intervention outcomes [2,5,15]. Core partnership practices include the implementation of democratic decision-making [16,17], the cultivation of collective empowerment [4,18], the incorporation of diverse cultural knowledge and multiple ways of knowing as essential aspects of community led health equity research [11,19,20], and building trust with communities [21–23].

Despite these advances, two major institutional gaps constrain the long-term successful development and conduct of federally and privately funded projects in addressing broader socio-political and economic conditions that produce health inequities [24]. First, there remain significant administrative barriers and insufficient policy development and support within academic health centers (AHCs) to create and sustain patient/community research beyond individual projects [25]. Despite community engagement cores or equity centers from multiple NIH funding sources, these barriers still include fiscal challenges in providing timely and sufficient community subawards, difficulties for IRBs to recognize community co-investigators, and lack of funding for sustainable partnerships, among others. Second, there is a need to enhance patient/community leadership to set their own research agendas for greater community autonomy and power both within and outside of academic institutions [26–29].

Engage for Equity PLUS (E2PLUS) sought to transform AHCs through supporting champion teams of academics, community, and patient partners to strengthen research infrastructures to support CBPR/CEnR. In this two-year Patient Centered Outcomes Research Institute (PCORI) engagement feasibility award reported here, E2PLUS scaled up the use of their E2 intervention tools previously tested with individual projects, and added new institutional change and patient/community support strategies to work with three diverse institutions: Morehouse School of Medicine (MSM), Stanford School of Medicine and Stanford Cancer Institute, and the Fred Hutchinson Cancer Center/University of Washington.

This paper uses a qualitative, exploratory case study analysis to describe how contextual conditions influenced champion team decision-making for strategies that created greater institutional engagement and with communities. For more on contextual conditions, see Adsul et al. 2025 [30]. We first present a brief background of the intervention, describe the settings and qualitative case study methods. We then analyze the engagement strategies that champion teams adopted to address specific contexts and conditions. Finally, we discuss the overall learnings and implications of E2PLUS reflection-action strategies for other AHCs working towards sustainable community/patient engaged research structures, policies, and practices.

### Intervention description

E2PLUS scaled up the previous Engage for Equity (E2) research of the University of New Mexico Center for Participatory Research, with national partners, which was funded by three National Institute of Health (NIH) studies since 2006. Through surveys of over 400 research projects and eight case studies, E2 produced a CBPR Conceptual Model, analyses of partnering best practices, psychometrically validated survey instruments for partnership evaluation, and E2 intervention workshops and tools to strengthen partnerships [4,31,32].

Given our institutional focus, we adapted historical institutional theories of change which posit that stakeholders are most likely to develop and pursue change strategies based on the historical, political, and structural conditions of their organizations [33–37]. These conditions are typically manifested in formal rules and policies related to research, institutional norms about what defines high-quality health research, the reputational identity of the AHC, and other material conditions such as funding sources, the size of the AHC, and the design of the research infrastructure.

Institutional conditions act as filters that inform goals and strategies that groups and leaders are willing to adopt for change. For example, leaders from AHCs in which only a small portion of research is dedicated to community engagement may be more likely to pursue incremental changes, such as increasing the number of CBPR faculty; compared to institutions with a strong community engagement history, which are more likely to pursue transformational strategies, such as integrating indigenous epistemologies as a cornerstone of center-based research initiatives[38]. The literature on institutional change also suggests that among AHCs with a diffuse network of leaders holding power in different areas (e.g., in departments, centers, schools, and campuses) institutional change is likely to be slower, more incremental, and require coordination and collaboration among multiple leaders with competing agendas compared to AHCs with concentrated authority. In short, as Wilkson and Alberti attest: leadership matters and advocating for change not only requires the support of leaders, but also interventions that consider how leaders must navigate their own institutional structures [39].

E2PLUS builds from a growing literature that underscores the need to support innovative CEnR practices, stronger co-governance to better support the complex interdependence of patients/community, health systems, research centers, and traditional academic units in creating dynamic inter-sectoral processes and practices that are more likely to change the social determinants of health and promote health equity outcomes over the long term [40–43]. A key innovation of E2Plus was to pragmatically identify conditions for change, understand contextual and systems diversity among AHCs, and support each site to develop attainable strategies based on their embedded institutional structures and discourses. Strategies included developing and implementing a range of practices to enhance collective capacity in CEnR research (e.g. creation of affinity groups, hiring community ambassadors) and convincing leaders to adopt organizational policies that support community power in research. A detailed description of E2PLUS’ intervention theory of change is published elsewhere [4,24].

## Methods

This study used an exploratory qualitative design to assess the feasibility of the E2PLUS engagement intervention to raise critical awareness of conditions supporting and inhibiting CBPR and P/CEnR and to describe the kinds of strategies champion teams crafted and executed to alter institutional policies, rules, norms, and procedures to increase support of P/CEnR and community power.

### Intervention setting

The team selected three institutions that were unique geographically and demographically, served different populations and varied by support for P/CEnR, in order to examine whether E2PLUS was feasible across different settings. While initially we expected the institutions to reflect similar constellations of an AHC, we quickly learned that we were working with three distinct organizational structures that also reflected the complex interdependence of research, health systems, communities, and health professionals in AHCs [43,44]

Morehouse School of Medicine (MSM) is a private, historically black college located in Atlanta. Started in 1975, with research uncovering that 75% of Georgia’s black population was being cared for by just 93 black physicians, MSM now continues medical education, with ongoing emphasis on research that promotes medical anti-racism, health equity, and community engagement. Housed in MSM, the Prevention Research Center (PRC) was established in 1998 and has supported most of the institution’s CBPR. A priority of the PRC is to establish a symbiotic community partnership through culturally- and racially centered projects, and through its Community Coalition Board governed by bylaws which assert community decision-making. E2PLUS was centered in the PRC. As an independent medical school with a strong commitment to health equity, its President and other top leaders would prove to be easily accessible to the champion team led by the well-recognized PRC.

Stanford is a highly ranked private university supported by a longstanding endowment. The Stanford School of Medicine has invested heavily in genomics, stem cell treatment, cancer predictive diagnosis, organ transplants, and precision medicine, with status in the last decade as having the highest National Institute of Health funding per SOM researcher in the country. Despite stark income inequalities in the region, community engagement efforts have only recently blossomed at Stanford when the current Office of Community Engagement (OCE) was started in 2018 with a small cadre of academics doing place-based work in the region and nationally. E2PLUS was centered in the Clinical Translation Science Award (CTSA) OCE, the Office of Cancer Center Health Equity, and SOM Department of Pediatrics. With multiple institutional entities, including IRBs located above the SOM at the University level, it would become apparent that the champion team would have to engage top leaders at different levels.

Since its 1973 National Cancer Institute (NCI) designation, Fred Hutchinson Cancer Center (Fred Hutch) had a history of well-recognized individually-led CBPR, with several external community engagement sites and strong patient advocates. Their comprehensive Office of Community Outreach and Engagement (OCOE) was recently established because of NCI requirements. Partnering with Fred Hutch was the University of Washington (UW) Institute of Translational Sciences and School of Medicine, as a public institution with high-level NIH funding. Simultaneously with E2PLUS, a Cancer Consortium merger was taking place between Fred Hutch, UW, Seattle Cancer Care Alliance and Seattle Children’s Hospital, which leveraged momentum for policy change. E2PLUS was centered in Fred Hutch OCOE with UW partners. While the merger provided momentum, the champion team would realize they needed to focus their advocacy with Cancer Center and CTSA top leadership.

Stanford represented the earliest in its development of a community-engaged research infrastructure, Fred Hutch/UW in the middle, with MSM as a nationally recognized CBPR/CEnR-oriented institution

### Intervention components

**Figure 1.**
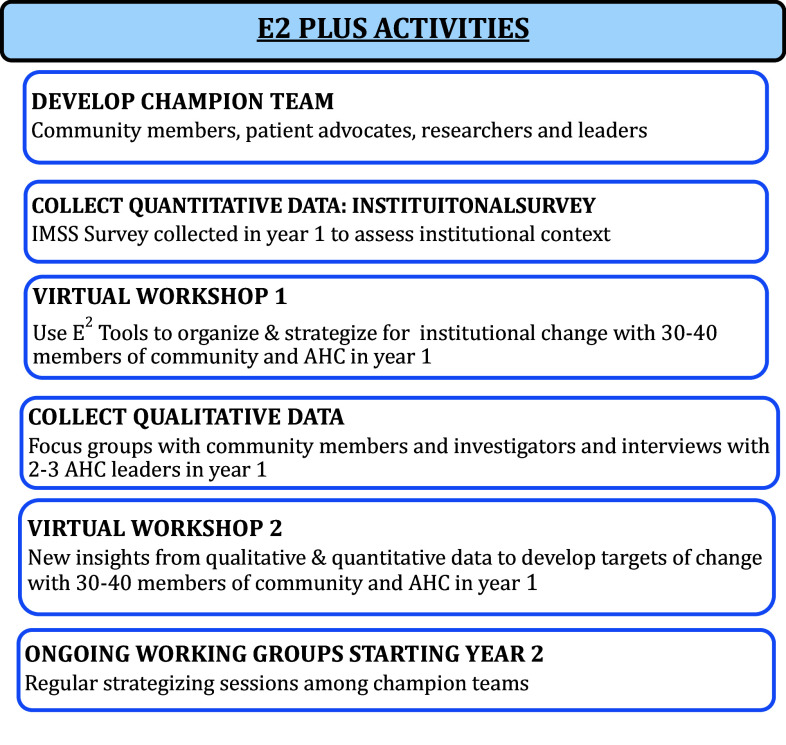
Core E2 PLUS intervention components.

As Figure 1 demonstrates, E2PLUS first year strategies were to 1) Conduct institutional assessments of barriers and facilitators (through surveys, interviews, and focus groups); 2) provide monthly bidirectional coaching and mentorship meetings for champion teams of 6–8 members comprised of P/CEnR researchers, engagement center leaders, patient advocates, and community leaders; 3) provide two reflection/action virtual workshops with E2 tools to a larger stakeholder group (with the first workshop having participants capture their own engagement context and histories, and in the second workshop identify desired policy changes and working groups); and 4) leverage data for champion teams and working groups to inform top leaders of current strengths and gaps and create advocacy for policy and practice changes.

With champion teams as the drivers of change, each was formed by a strong mid-level leader, i.e., the directors of their community engagement offices or the PRC, who invited their key partners. The Fred Hutch OCOE director mostly included colleagues from Fred Hutch and UW SOM, Stanford’s team included their CTSA, their Cancer Institute, and Pediatrics, and Morehouse had members and partners from their PRC. Each also invited core community partners to participate. The UNM E2 team had relationships with each of these mid-level leaders who were excited to join, but their top leaders had no prior knowledge of the project. They would soon learn from champion team activity and from targeted top leader interviews.

The second year was driven by the champion team and working groups setting their own timelines, with the UNM team participating as reflection partners. Year two activities leveraged data from qualitive interviews and focus groups and data from the quantitative E2PLUS Institutional Multi-Institutional Stakeholder Survey to assist each champion team in planning strategies for change [45]. We expected that ongoing coaching and co-analyses of data would enable champion teams to clarify their histories of engagement, identify tensions within their contexts, deepen critical consciousness of their conditions, and see opportunities for change strategies, which could differ based on conditions. Finally, E2PLUS hosted quarterly community of practice (COP) meetings between all sites to share frustrations, successes, and mutual learnings that we hoped would further national organizing for policy changes among top AHC leaders and funders.

### Data collection

The UNM team conducted participant observation, focus groups, and interviews, with purposive sampling to recruit participants. For each site, in the first-year in-between workshop one and two, focus groups were conducted: one with AHC researchers and research staff, one with patients and community members associated with the AHC, and one with the champion team. At the same time, 3–4 interviews were conducted with top leaders. Champion teams also had a focus group at the end of the two-year intervention. Table 1 summarizes the qualitative demographics.

**Table 1.** Participants in qualitative data collection

	Institution *n* = participants		
**Data Collection** **Method**	Morehouse School of Medicine	Stanford School of Medicine	Fred Hutch/ University of Washington
**Leader Interviews**	3	4	4
**Focus Groups**			
Patient/ Community	10	4	8
Investigator	1	4	4
Champion Team	9	11	18

Other qualitative data w collected at workshops, with the River of Life [32,46] used for capturing engagement histories; and visioning with the CBPR Model [32,47] used to capture strategic planning targets for change monitored over time.

We designed semi-structured guides for leader interviews and focus groups. Leader interviews included questions about institutional barriers or facilitators for supporting P/CEnR policies and practices. Investigator focus group guides had questions regarding perceptions on the institution’s value of P/CEnR and CBPR, policies and practices acting as facilitators and/or barriers, and changes that could be made to strengthen patient and community engagement. The patient and community guides included additional questions on power dynamics. See supplemental file 1 for sample questions.

### Data analysis

Interviews and focus groups were audio-recorded and transcribed verbatim. Additionally, field notes from workshops, champion team meetings, and working groups were recorded and used. The team developed the codebook based on question-levels that guided the initial analysis. Identifying information was removed from the transcripts before distributed to the research team for review and analysis. All interviews were recorded and transcribed with all identifying information removed. Transcripts were imported in ATLAS.ti 10 to facilitate organizing, coding, searching, and retrieving data.

## Results

Results are reported by 1) the development of critical consciousness of contexts; 2) the adoption of change strategies by differing institutional contexts; and 3) by impact of COP.

### E2PLUS stimulates critical consciousness of institutional conditions

Early in E2PLUS, the first workshop (which took place within the first four months) was a critical juncture for thinking about institutional change. Thirty-five to forty participants (including investigators, community members/patients, and a few top leaders) invited by the champion team engaged in the River of Life exercise, which provided a space for the larger stakeholder group to craft a shared knowledge base about their institution’s history of patient and community engagement. This opportunity for a multi-sector coalition started the signaling to leaders that there could be broader momentum for structural changes. Workshop one also enabled participants not only to recognize their national and local strengths but also their institutional limitations in CBPR/P/CEnR. Though champion team members may have had some of this understanding in earlier meetings, the opportunity to hear stories, and reflections strengthened their understanding of their own conditions.

The “River of Life” activity cued champion team participants to learn of and document their institutional history, priming them to utilize their knowledge of the past to craft engagement strategies that would address contextual conditions and alter the landscape of their institution’s river:

I have heard a little bit about that history, but it seemed to really crystallize more hearing people in those meetings where a lot of the prior community engagement efforts were service delivery medical education…. We have more awareness now for what we are trying to do and research aspects and the CBPR components which are different to that. The challenges are in infrastructure and resources that we need for this… (Stanford Champion Team FG)

These realizations partly occurred because the workshops included individuals with varying roles, knowledge, and involvement within the institution:

One thing that impressed me [is that] we invited folks who had been around Stanford for a long time, and I think everyone on this call is really new—and so the history that we share together is pretty recent. I gained a deeper appreciation of what has been seen as community engagement over decades… and it seemed to me that most of it was related to clinical care… (Stanford Champion Team FG)

Workshop one also helped champion teams realize the benefit of uniting as a champion team to forge ahead on identifying targets of change. One investigator from MSM noted that the workshop allowed for time and space in everyone’s schedule to simply come together as one important benefit of the intervention activities,

The workshop was good, because we don’t always get a chance to stop and think about ourselves. We can think about what we’re doing well and what we can do better. All of us need to have this protected time to stop and think about it (Morehouse Champion Team FG).

At the end of the first year, the UNM E2PLUS team compiled the data from workshops, meeting observations, surveys, focus groups, and interviews for a mixed methods analysis to begin to identify contextual conditions that clarified differences as well as commitments to community engagement and opportunities for change. As table 2 indicates, this emerging typology included: each institution’s identity and national reputation; the type and extent of national funding as a core external force that fed the national reputation; their history of community and patient engagement; their local conditions and events during the intervention; and their backbone center which hosted their champion team for participating as a partner with the E2 PLUS intervention. Exploratory analyses revealed that in spite of different contexts, each site shared similar conditions that impacted support for P/CEnR.

**Table 2.** Contextual conditions. Summary of the contextual conditions identified across institutional sites

	Stanford School of Medicine	Fred Hutch/UW	Morehouse School of Medicine
**INSTITUTIONAL IDENTITY**	Early Endowment from Railroad money, called “The Farm” Elite Private West Coast with access to large network of Silicon Valley to foster innovators and entrepreneurs	Working toward the formation of a Cancer Consortium National Institute of Health & Pharmaceutical money along with and local Philanthropy. (i.e., Bill and Melinda Gates Foundation and Amazon) Top Tier Public University with national recognition of the university and cancer center	Developed from connection to Black Community Private Historically Black College & Universities (HBCU) School of Medicine Leaders of national Prevention Research Center (PRC) network and Leader of National Comprehensive Cancer Network (NCCN) as community partners of the PRC National recognition as health equity and CBPR leader based on “Morehouse Model”
**NATIONAL EXTERNAL FUNDING**	Prominence of biomedical research historically Very high research activity (R1) Basic Science, Genomics, precision medicine that is moving into clinical research and beginning place-based, Part of California NIH Community Engagement Alliance Clinical and Translational Science Award (CTSA) Cancer Center Support Grants (CCSGs) for National Cancer Institute-designated Cancer Centers	National Cancer Institute (NCI) new mandates for Office of Community Outreach and Engagement (OCOE) Very high research activity (R1) Basic Science and clinical research, with place-based cancer catchment area and with isolated community research	Long-term Prevention Research funding with community engagement focus Rapid growth of place based and CBPR funding during COVID: National Hub for National COVID-19 Resiliency Network (NCRN), Rapid Acceleration of Diagnostics (RadX), and NIH Community Engagement Alliance (CEAL)
**LOCAL CONDITIONS AND EVENTS**	Histories of clinical outreach but not community research Administrative burden with medical school under university bureaucracy	Recent individual community- engaged research (CEnR) Racism charges at Seattle Children’s and at UW–led to widespread strategies for anti-racist policies/practices Vocal student community	Need to expand Community Coalition Board (CCB) with population changes in Georgia to grow Latino representation and participation in research.
**HISTORY AND DEGREE OF COMMUNITY ENGAGEMENT**	Individual faculty had support from their chairs to pursue principal investigator (PI) led CBPR projects with individual Community-Based Organizations (CBOs)	Fred Hutch started with a single committed community-engaged researcher who provided the foundation for the later National Cancer Institute requirements to expand the OCOE to expanded engaged research with coverage of the catchment area as the entire state.	Strategic Plan (developed in 2015) brought community engagement as the fourth pillar with a dashboard of accountability Prevention Research Center (PRC) core projects of many years (established 1998) Core Morehouse team with Community Coalition Board as decision-making partners
**BACKBONE CENTER(S) TO CHAMPION TEAM**	Clinical and Translational Science Award (CTSA) Community Engagement Core Academic Departments, e.g., Pediatrics Office of Community Outreach and Engagement (OCOE) at Cancer Center	Fred Hutch Office of Community Outreach and Engagement (OCOE) UW School of Medicine, Institute of Translational Health Sciences (i.e., CTSA) Emerging UW Fred Cancer Consortium	Prevention Research Center (PRC) with Community Coalition Board (CCB) community power Morehouse School of Medicine

### E2 PLUS champion team strategies to inform and shape institutional changes

In the second workshop of year one, the E2 team provided a preliminary summary of the mixed methods contextual analysis, including tensions that potentially hindered or supported each site to strengthen community engagement infrastructures. The workshop visioning exercise with the CBPR Model then incorporated this institutional analysis for co-interpretation to both identify strategies for advocacy to top decision-makers and to increase social and political connections between faculty/staff and community members/patients.

In the final year one report, E2PLUS data were provided to the champion team for further co-analysis and coupled with subsequent coaching of champion team played a critical role in shaping champion team actions based on their local conditions, histories, and institutional designs. Reported below is an analysis of each site’s strategies based on their contexts.

### Morehouse school of medicine

As a Historically Black College and University, MSM has a long history of successful research and practice to promote health equity, particularly among the African American community, with top administrators declaring that “equity is in our DNA.” Codified health equity principles include, for example, MSM’s 2017 strategic plan adoption of community engagement as a fourth pillar with accountability measured annually across departments and offices. In 2020, the MSM PRC published “The Morehouse Model” of health equity and community engagement best practices, prioritizing capacity-building for community leaders and researchers [48].

MSM’s Prevention Research Center (PRC) has been recognized as a national community engagement leader with a track record of institutionalized power-sharing in research within the local African American community via their Community Coalition Board (CCB) [48]. The CCB was led by and primarily comprised of community members, enabling community members to shape the PRC strategic direction, to share decision-making authority with academic leaders and to directly influence funding priorities. Through participating on MSM’s IRB, community PRC members also influence the direction of research ethics and prioritization of community benefit. During COVID, the MSM received major NIH funding through the National COVID-19 Resiliency Network, RaDx® Underserved Populations, and Community Engagement Alliance against COVID-19 Disparities, of $40 million dollars, and the director of the PRC assumed additional leadership positions. These external funds have strengthened their capacity to move beyond simply checking the box of community engagement to cementing patient- and community-engaged research within MSM’s overall mission.

Despite these strengths, E2PLUS workshops and data analyses revealed institutional challenges. For example, leaders and researchers identified a shortage of human resources necessary to sustain a wide-reaching community engaged infrastructure because a small cadre of leaders primarily fulfilled this role. Second, further work was needed to ensure that health equity was grounded in all MSM activities, from institutional planning, basic research science, education and training of students, to dissemination of evidence-based knowledge in the community.

Several participants noted the intransigence of racism as a roadblock for deeper codification of health equity values within MSM. From a community member’s perspective, racism is “so emotionally-laden” that one way to navigate is to choose not to talk about it. PRC leaders also said that there were subtle pockets of resistance to health equity approaches evidenced by the demeanor of many leaders wanting to appear “neutral and nice” as opposed to addressing systemic inequities, that are deeply ingrained.

Dialogue at both E2PLUS workshops also revealed some challenges related to the PRC governing board (CCB). For example, newer community leaders discussed a power imbalance that stemmed from the absence of a formal process for on-boarding new community members to the CCB. Others noted research-related challenges, with CCB community member stating that “the CCB feels disconnected” from the actual results of research undertaken at MSM. Finally, board leaders and members acknowledged the need to expand their stakeholder base to include other groups such as the growing Latino population. Another participant shared that it was not easy to link institutional policies and practices in MSM with the PRC because “the highest level has a different floor” implying that there is a lack of coordination and consistency across the institution with respect to formalized processes for garnering community input and governance.

Despite these challenges, MSM demonstrated substantial institutionalization of health equity-based community engagement in research and clinical practice. E2PLUS created the space and time for community, patients, and academics at MSM to ideate and discuss the current barriers and strengths within their institution. E2PLUS also brought new partners and collaborators interested in CBPR/CEnR research into the loop, breaking down communication siloes. As a result, the intervention allowed the champion team to focus on strategies which enabled the *maintenance* and *strengthening* of their successes. For example, the champion team identified greater acknowledgement of community/patient partners in formal agreements with MSM and reaffirmed their goal of creating avenues of power for community and patient organizations to receive funding independently. Yet, they also focused on expanding efforts to include additional stakeholder groups in their work.

### Stanford school of medicine

Leaders, faculty investigators, and community members agreed that while Stanford maintained a strong reputation for research excellence in basic science and precision medicine, Stanford was often inaccessible and disengaged from local, place-based communities that experience economic, racial, and other inequities. For example, an institutional leader at Stanford stated that Latino and African Americans distrust the institution because most research does not directly benefit the community. Community stakeholders echoed this sentiment and added that some of this disconnection stems from a perceived lack of representation of underrepresented minoritized groups among student, staff, and faculty. Compared to MSM, Stanford demonstrated less codified adoption and implementation of health equity principles into research practice and lacked widespread institutionalized power sharing practices to conduct research with local place-based community organizations.

Prior to E2PLUS, some department chairs and center leaders within the MSM began to recognize the need to expand Stanford’s focus on basic science research to clinical research and to a certain extent, P/CEnR and CBPR. Though much of this outreach started with national community engagement mandates from NCI and Clinical Translational Science Awards, a small cadre of academics also expanded their research beyond clinical settings to place-based efforts linking health equity interventions with underlying living conditions impacting health among poor and communities of color. These faculty from multiple departments incubated many project-based initiatives with strong reliance on individual relationships between faculty and community leaders.

Despite these advances, the contextual analysis revealed two major roadblocks. First, while there were a handful of strong principal investigators conducting patient and community engagement research, community engaged work was organizationally fragmented. Research investigators expressed frustration that “everyone is doing this in their bubble” without clear protocols or understandings of broad “community needs and interests.” Second, given the complexity and commercial implications of much of Stanford research, champion team members identified the need to improve Stanford’s bureaucratic processes. Center directors and department chairs were particularly vocal about structural constraints that emanated from working in “a highly decentralized, highly distributed institution with many research and administrative silos.”

The champion team created a collective space for a group of newly emerging leaders in P/CEnR to unify efforts through generating a common policy agenda. Specifically, they leveraged the E2PLUS data analysis to inform leaders of problems they were unaware of and harnessed their participation in other preexisting efforts to build a broader internal coalition to pressure leaders to address organizational challenges. Consequently, the champion team formed two working groups: one, to advocate for restructuring IRB protocols to facilitate efficient and effective processes for P/CEnR; and two, to work with leaders to streamline SSM and university post-award policies and procedures for contracts and payment to community partners.

Additionally, leaders and community members stressed the need to “define multiple layers” of what constitutes community representation and participation in research. One leader raised the challenge that the field needs to specify how different P/CEnR approaches should be employed with different types of partners to provide effective institutional support. Community stakeholders agreed, but they focused more on the necessity to differentiate between competing and sometimes opposing interests of community stakeholders representing government institutions, grassroots activists, community members, patients, and community-based organizations. Importantly, this included acknowledging power differentials between stakeholders.

Champion team leaders also used the intervention to increase collective capacity in P/CEnR and leveraged CTSA resources to create a new program hiring community leaders as Health Equity Ambassadors within and outside of Stanford. The Ambassadors would not only strengthen community access to Stanford but would provide a bridge between place-based community organizations with emerging clinically based P/CEnR researchers. Finally, a champion team leader recognized the necessity to move from supporting vibrant individual P/CEnR researchers and community leaders to redesigning center-based community advisory boards to strategically include CBOs and community leaders who were positioned to create healthy-bonding relationships within historically marginalized communities and to foster a stronger bridge across academics and CBOs. Indeed, a core innovation of this champion team was to depersonalize CBPR practices by moving away from individual power toward striving to create community co-governance with stronger community advisory boards and collective capacity building practices.

### Fred hutch/university of Washington cancer consortium

As described above, the first year of engagement with E2PLUS overlapped with a merger among Fred Hutch, Seattle Children’s Hospital, Seattle Cancer Care Alliance, and UW Medicine. This merger provided opportunities for proposing new shared policies and practices that could include prioritizing patient perspectives.

The data analysis however revealed ongoing procedural l barriers, including lack of overarching structures for community engagement, grant deadlines not being conducive for community engaged research, and a lack of time and effort exerted into building partnerships within the AHC. Like Stanford, several participants highlighted that individual faculty members were seen as P/CEnR resources, but there were few sustained patient/community workstreams, which would include needed templates and resources for specific research tasks. Unlike MSM, these participants revealed shortcomings in successfully administering research to support sustainable CEnR.

Similar to Stanford, workshop two and focus groups also generated substantial discussion around how the consortium faculty lacked a shared vision of the purpose and goals of P/CEnR. Some stakeholders primarily saw P/CEnR as a short-term return on investment through increasing minority recruitment into clinical trials, or stated they still needed evidence that P/CEnR contributes to better science. Others viewed engagement as a long-term process that required building relationships for authentic partnerships for improving equity. There was significant skepticism among current committed investigators and patient advocates of how P/CEnR was valued outside of existing practitioners:

In these processes they need to stop having predetermined outcomes…if you ask me to be on a grant don’t take your cookie cutter formula and stick it in there and [ask me] to check the box (Fred Hutch Patient FG).

These differing worldviews suggested that in order for institutional change to occur, it was necessary to move beyond public health/community scientists practicing CBPR to promoting the adoption of P/CEnR among other clinical/basic scientists.

Finally, while several faculty and leaders said there was good forward movement, patient advocates and community members felt there were still gaps in engaging patients and community in research. For example, patient stakeholders expressed concerns with unresolved inequities in access to and quality of patient care, contributing to the view that community voices were not present or heard within the research enterprise.

The newly emerging cancer consortium presented a window of opportunity for the champion team to advocate for reforms to center *patient* engagement in research. For example, after the first E2 Workshop crystalized stakeholder support for institutionalizing P/CEnR, the faculty leader of the champion team immediately presented the workshop dialogue and E2PLUS intervention to Cancer Center leadership as an agenda-setting strategy to better integrate P/CEnR principles and practices into the organization.

The champion team was also a catalyst for promoting engagement that could be more accountable to patients. Importantly, cancer patient advocates on the champion team advocated to create a new Hutch Office of Patient Engagement (HOPE), in addition to their OCOE, that would serve as a platform to pursue advocacy, research, and information-sharing with the broader patient community. Patients viewed this office as a way to increase the collective capacity of patients to influence change and create stronger institutionalized power sharing between patients, academics, and staff. As a result of participating with the champion team, the Director of OCOE included other staffing needs in the following year budget request. While the functions of HOPE ended up being folded into another patient support office without the comprehensive focus, these specific advocacy efforts can become a model of how groups like these are important for incremental policy reform and for adding specific integration of patients into typical community engagement centers.

### Creation of a Cross Site Community of Practice

E2PLUS’ final aim was to establish a COP with regular virtual meetings in both years of the project. A final face-to-face meeting in New Mexico brought together a larger group of institutional champion team members for shared analysis of learnings. Champion teams from all 3 sites discussed the importance of the intervention, which provided a unique opportunity for critical conversations about the role of patients and community members in research and similarities and differences in institutional practices and policies at each institution.

Discussion emphasized the power of data to understand their own institutional conditions, and its importance for gaining allies within the top leadership. Community and patient partners also stated that data needed to be owned by the community, with data sharing and ownership agreements written into research plans.

If [patient and community members] are learning to ask the question and engaging in the design and outreach, you’re building capacity within that community to become your community scientist or next research prodigy who goes on to colleges and comes back and makes a difference in your communities. (COP Meeting)

This COP discussion suggested that true institutional change should be simultaneously catalyzed inside AHCs and outside the walls of the academy. Other common themes included the necessity to build strong sustainability for long-term P/CEnR work and the necessity to include new patient and community partners in institutional change efforts. Community leaders stressed prioritizing networking and bi-directional knowledge-building, with workshops led by patients/community members.

This meeting strengthened the network for peer support that went beyond institutional silos and allowed for broader conversations, stressing the importance of patients and communities in developing their leadership and priorities in research.

## Discussion and conclusion

E2PLUS demonstrated that progress toward institutional change that supports authentic and effective CEnR is possible when leaders and champions leverage local, contextual, and institutional data to create tailored strategies for change; when partners use ongoing collective reflection practices that generate critical consciousness about how the underlying organizational climate, policies, and conditions hinder or bolster equitable engagement; and when champions systematically collaborate with community and academic representatives to design new initiatives or policies that prioritize community preferences across a variety of health equity issues.

As the descriptive analysis suggests, creating champion teams with reputable, mid-level leaders from well-established centers within AHCs was a key factor in recruiting top leaders to participate in the intervention. Champion team leads also played a pivotal role in steering each teams’ efforts to build collective capacity for P/CEnR by promoting more pathways for shared governance and collaborating with strong patient and community advocates who were able to reinforce linkages between place-based communities and community engagement offices. These middle-level leaders provided an important bridge between AHC and community leaders and served as primary brokers working across diverse identities and interests of groups in P/CEnR. More longitudinal research is needed to more fully understand how community leaders and other players, such as health equity ambassadors, can engage systematically with feedback loops for sustained improvement in policies and practices supporting CEnR in AHCs.

Our analysis also suggests that the E2PLUS intervention enabled participants to leverage workshops, storytelling activities, and regular champion team meetings to shift preexisting relationships while creating new ones. These relational changes catalyzed champion teams to advocate for and create winnable actions. Importantly, the intervention revealed the critical role of patients/community members in the champion team and in their own dialogues, stating what one called, “non-negotiable demands” for changes in AHCs. The intervention also highlighted the important role of continual collective reflection, networking, and sharing successful initiatives across sites in the COP.

Finally, E2PLUS demonstrated the importance of using data and collective reflection practices for identifying and acting on contextual conditions that were likely to influence effective CEnR. These conditions include: 1) institutional identity and national reputation; 2) national funding as a core external force that feeds national reputations; 3) histories of community and patient engagement; 4) local conditions and events during the intervention; and 5) the role of the backbone center for champion team participation with the E2PLUS intervention. While Adsul et al. provided a mixed-method aggregate analysis of AHC contexts across sites [30], the results here showcase the importance of understanding how participants leveraged their specific contextual conditions to develop tailored strategies for institutional change. We also demonstrated the feasibility of scaling up E2PLUS tools and workshops, produced for individual projects, to the institutional level, with the additional strategy of supporting champion teams in bidirectional reflection and actions.

The analysis also revealed some limitations. MSM had already codified many CBPR/CEnR practices, so E2PLUS mainly gave them the space to reflect and maintain strong practices compared to other sites. This suggests that the intervention has potential ceiling effects and may be more suitable for AHCs still strengthening CBPR/CEnR institutional practices and policies. As an exploratory qualitative study, more research is needed to test the overall effectiveness of the intervention across multiple settings by implementing a standard intervention with a pre/post or modified RCT design.

Finally, across all sites, community participants said that while useful, champion teams primarily advocated for internal AHC policy changes. They called for future intervention research to leverage AHCs to promote independent venues for community-leaders and organizations to generate changes in research practices that promote greater community control of research. Building from this finding, E2PLUS’ new PCORI Science of Engagement award added a separate patient/community action group, supported by coaching from a national network of community leaders for enhanced decision-making and governance in local communities.

While the process continues, we found that champion teams leveraged E2PLUS to challenge institutional arrangements that would allow community more equal access to decision-making, develop more egalitarian approaches to knowledge production, and support stronger social connections and reciprocity between academic elites and communities to bridge differences and promote power sharing. The overall process allowed the E2PLUS team as an external group to assist each champion team in holding up a mirror to ACH’s and their leaders recognize, reflect on, and devise intuitional changes necessary to more sustained P/CEnR research.

## Supporting information

Sanchez-Youngman et al. supplementary material Sanchez-Youngman et al. supplementary material

## Supplementary material

The supplementary material for this article can be found at https://doi.org/10.1017/cts.2025.51.
