# Supplementary material for: Engage for equity plus: Transforming academic health centers to sustain patient/community engaged research structures, policies, and practices
Source: J Clin Transl Sci. 2025 Mar 19;9(1):e80. doi: 10.1017/cts.2025.51 (PMC12083203; doi:10.1017/cts.2025.51)
Supplement: Sanchez-Youngman et al. supplementary material [file S2059866125000512sup001.docx]

**Supplemental Table 1 List of Sample Questions.** Sample questions from the semi-structured focus groups and leader interviews.

| **Data Collection Method** | **Questions** |
| --- | --- |
| **Leader Interviews** | - What are the key facilitators that are promoting and sustaining equity-based patient/community engaged research in your institution? - What role, if any, do community or patient stakeholders’ currently play in overall governance, decision-making and agenda setting in your institution? - What changes would you like to see happen in the next year or so to strengthen equity-based patient and community engagement as a sustained committed effort? - How do you envision institutional transformation or innovation for your university? What are some of your key initiatives? What are your key priorities? |
| **Focus Group** | - How do you perceive the institution values community-based participatory research and patient-engaged research? - How does the institution show they value community-based participatory research and patient-engaged research? Or how do they not? - How do you think community-based participatory research and health equity research would look different if communities were driving it? - What changes, if any, do you see are needed in the institution to strengthen community/patient engagement as a sustained committed equity-based effort? |
